# Decreased Na^+^ influx lowers hippocampal neuronal excitability in a mouse model of neonatal influenza infection

**DOI:** 10.1038/srep13440

**Published:** 2015-08-27

**Authors:** Hoyong Park, Ji Eun Yu, Sungmin Kim, Sang-Soep Nahm, ChiHye Chung

**Affiliations:** 1 Department of Biological Sciences, College of Bioscience and Biotechnology, Konkuk University, Seoul, 143-701, South Korea; 2 Laboratory of Veterinary Anatomy, College of Veterinary Medicine, Konkuk University, Seoul, 143-701, South Korea

## Abstract

Influenza virus infection is one of common infectious diseases occurring worldwide. The human influenza virus can infect the central nervous system and cause brain dysfunctions affecting cognition and spatial memory. It has been previously shown that infection with the influenza viral protein within the hippocampus decreases Ca^2+^ influx and reduces excitatory postsynaptic currents. However, the neuronal properties of animals surviving neonatal infection have not been investigated. Using a mouse model of neonatal influenza infection, we performed thorough electrophysiological analyses of hippocampal neurotransmission. We found that animals surviving the infection exhibited reduced spontaneous transmission with no significant defects in evoked neurotransmission. Interestingly, the hippocampus of the infected group conducted synaptic transmission with less fidelity upon repeated stimulations and failed to generate action potentials faithfully upon step current injections primarily due to reduced Na^+^ influx. The reversal potential for the Na^+^ current was hyperpolarized and the activation of Na^+^ channels was slower in the infected group while the inactivation process was minimally disturbed. Taken together, our observations suggest that neonatally infected offsprings exhibit noticeable deficits at rest and severe failures when higher activity is required. This study provides insight into understanding the cellular mechanisms of influenza infection-associated functional changes in the brain.

Influenza A virus is known to be capable of infecting the central nervous system in animal models^1^,^2^ and is reported to cause neurological disturbances^2^ and neuropsychiatric disorders in some occasions^3^. Influenza-associated neurological complications such as febrile/non-febrile convulsion, seizure, and altered consciousness have been reported in numerous clinical cases^4^,^5^,^6^,^7^,^8^,^9^,^10^,^11^,^12^. In particular, a recent European case study reported that three out of five children exhibited persistent impaired cognition, decreased social skills, memory deficits, and executive function after recovery from influenza infection, which are common symptoms observed in patients with neuropsychiatric disorders^7^. Studies using animal models have demonstrated that the human influenza virus (H1N1) and highly pathogenic avian influenza virus (H5N1) can infect extensive brain regions and subsequently cause neuronal cell death^1^,^2^,^13^,^14^. These changes have been shown to cause neuroinflammation accompanied by glial activation and altered proinflammatory cytokine expression^1^,^2^,^13^,^14^. Interestingly, different research groups have reported that influenza virus infection may have contributed to neurological or neuropsychiatric disorders in experimental animals^15^,^16^,^17^,^18^. For example, mice pups born to an influenza-infected dam exhibited autism- and schizophrenia-like behavioral disturbances^15^. Furthermore, abnormal behaviors in mice pups after maternal influenza infection have been reported to be accompanied by schizophrenia-like changes in serotonin and glutamate receptor expression in the frontal cortex^16^. In addition, previous studies have suggested that influenza virus infection induces abnormalities in synaptic function^19^,^20^,^21^. A series of studies have demonstrated that maternal influenza infection can alter the expression of myelination-related genes^18^,^22^,^23^, some of which have been identified as candidate schizophrenia genes^24^. The transduction of viral nucleoproteins (NP) into primary hippocampal neurons as well as influenza infection have been shown to decrease spontaneous excitatory activity and reduce Ca^2+^ currents in cultured hippocampal neurons^19^,^20^. However, a direct examination of the influenza virus infection on synaptic transmission has not been investigated. Here, we aimed to examine functional changes in the hippocampus at the synaptic level upon influenza infection by taking advantage of a mouse model of neonatal H1N1 viral infection^25^. We found that the surviving mice exhibited impaired neurotransmission as well as decreased neuronal excitability which was mainly due to the impaired activation of Na^+^ channels. We therefore performed a thorough analysis of the kinetics of the Na^+^ currents. We observed that the activation of the Na^+^ channels was slower and the reversal potential of the Na^+^ currents was hyperpolarized in the infected group, which could lead to deficits in brain function after neonatal influenza infection. Our results provide useful insight into the influenza infection-associated functional changes that have been observed in previous studies.

## Results

### Neonatal influenza infection causes impairments in the basal activity of hippocampal neurons

**Figure 1:**
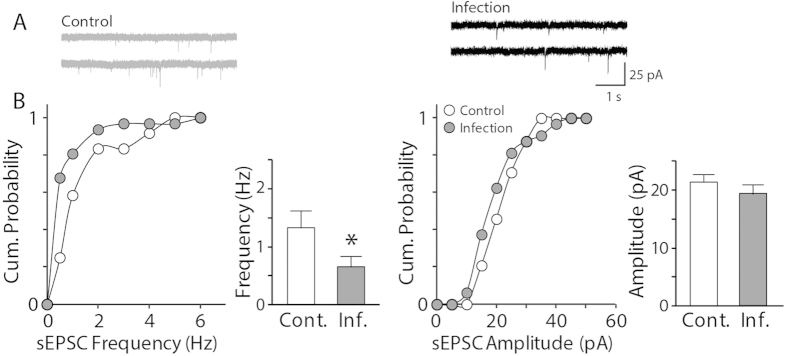
Hippocampal neurons exhibit decreased spontaneous neurotransmission after neonatal influenza infection. (**A**) Representative recording traces. (**B**) The frequency of spontaneous excitatory postsynaptic currents (sEPSCs) was decreased in the hippocampal pyramidal neurons of the mice that survived the neonatal influenza infection (n = 24–31, *p < 0.05), whereas the amplitude of the sEPSCs remained comparable to the control group (p > 0.3).

To examine whether neonatal viral infection causes alterations in the basal synaptic transmission of hippocampal CA1 neurons, we measured spontaneous excitatory postsynaptic currents (sEPSCs) from individual pyramidal neurons using whole-cell patch-clamp recordings in an influenza-infection model in neonatal mice. We observed a decrease in the frequency of sEPSCs in the infected group compared with the non-infected control group (Fig. 1, p < 0.05), whereas there was no difference in the amplitude of sEPSCs between the two groups (p > 0.3). This observation suggests that the basal synaptic efficacy is slightly but significantly impaired after neonatal influenza virus infection.

### Neonatal influenza infection causes impairments in the evoked neurotransmission of the hippocampus in an activity-dependent manner

**Figure 2:**
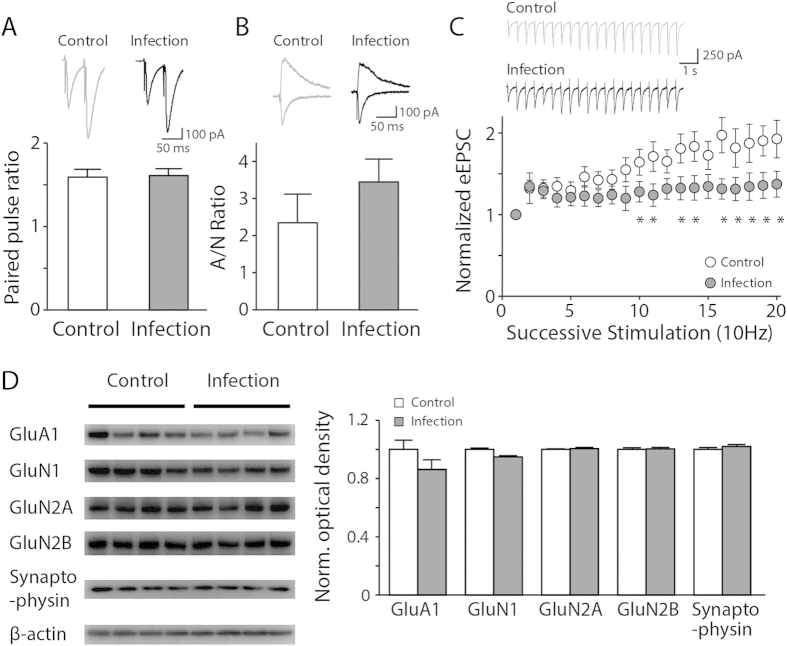
Synaptic transmission is less reliable in infected mice than in uninfected controls upon repeated stimulations. (**A**) The paired-pulse ratio of the evoked excitatory postsynaptic currents (eEPSC) by 50-ms interval electrical stimulations exhibited no significant change in the infected group compared with the uninfected controls (n = 13–15, p > 0.8). (**B**) The ratio of AMPAR-mediated eEPSCs at −60 mV and NMDAR-mediated eEPSCs at +40 mV was not altered in the hippocampus of the infected group (n = 7–13, p > 0.2). (**C**) The eEPSCs of the hippocampal neurons from the control and influenza-infected mice in response to 20 consecutive stimuli (10 Hz) revealed a decreased amplitude of eEPSCs upon successive simulations in the infected group as compared with the control group (n = 5–8, *p < 0.05). The insets show representative traces (gray: uninfected control group, black: infected group). (**D**) Representative images and quantification data of the western blot analyses for GluA1, GluN1, GluN2A, GluN2B, and synaptophysin in the mouse hippocampus at 21 dpi. The expression of GluA1, GluN2A, GluN2B, and synaptophysin remained comparable to controls (n = 4, p > 0.3–0.8), and a tendency of a slight decrease in GluN1 expression was observed in the hippocampi of the infected group (n = 4, p = 0.0571).

In order to investigate any possible defects in the activity-dependent neurotransmission in the hippocampus of the mice that survived, we measured evoked EPSCs (eEPSCs) from the hippocampal pyramidal neurons while stimulating the Schaffer collateral pathway with a bipolar stimulator. When two consecutive stimulations were given in a pair with a very short time interval (50 ms), we observed no significant changes in the ratio of the amplitude of the two eEPSCs evoked by each stimulation between the control and neonatally infected animals (Fig. 2A, p > 0.8). This observation indicates that there were no significant alterations in the presynaptic release probability of the neurons after neonatal infection. To examine infection-mediated postsynaptic changes, we compared the relative amplitudes of AMPAR- and NMDAR-mediated eEPSCs. We found no significant difference in the ratio of AMPAR- vs. NMDAR-mediated eEPSCs, suggesting no primary postsynaptic deficits upon neonatal influenza infection (Fig. 2B, p > 0.2). However, when these neurons were challenged with a series of stimulations, such as 20 successive stimulations at 10 Hz, the infected group exhibited less synaptic transmission as the number of stimulations increased compared with the control group (Fig. 2C, p < 0.05).

Next, we examined whether the proteins essential for reliable synaptic transmission exhibit any changes in their expression after neonatal influenza infection. The expression levels of GluA1, GluN2A, GluN2B, and synaptophysin in the hippocampus of the infected group were comparable to those in the control group (Fig. 2D, p > 0.3). GluN1 showed a tendency of a slight decrease in the neonatally infected group compared with the control group (Fig. 2D, ~95% of controls, p = 0.0571); however, we did not detect any functional changes in the electrophysiological recordings (Fig. 2B).

### Neonatal influenza infection decreases hippocampal excitability

**Figure 3:**
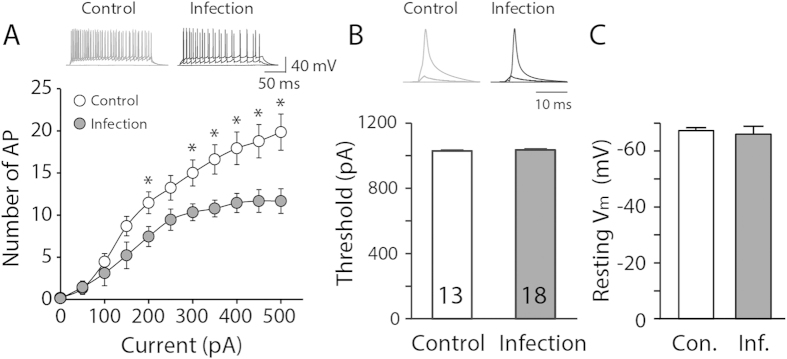
Neonatal influenza infection decreases neuronal excitability of the hippocampus. (**A**) The number of action potentials in infected mice significantly decreased in response to the given depolarization steps (250-ms duration, 50-pA increment, 11 steps). The injection of a strong depolarization current (>200 pA) evoked less action potentials in the infected group compared with the uninfected control group (n = 15–20, *p < 0.05). (**B**,**C**) The threshold to initiate action potentials or the resting membrane potential in the infected group (Inf. or Infection) remained comparable to the control group (Con. or Control) (p > 0.4, n = 9–13 for the threshold; p > 0.6, n = 13–18 for the resting membrane potential).

Given our observation of selective deficits in an activity-dependent manner, we further investigated the electrical properties of hippocampal neurons after neonatal influenza infection. When we quantified the number of action potentials upon 500-ms depolarizing current-step injections (50-pA increment), we observed reduced firing of action potentials in the neonatally infected animals (Fig. 3A, p < 0.05). The threshold current size for initiating action potentials was comparable between the groups (Fig. 3B, p > 0.4) and the resting membrane potential between the two groups of animals was not significantly different (Fig. 3C, p > 0.6). This observation provides further support for our previous results with consecutive high frequency challenges, suggesting that the hippocampus of infected animals performs poorly in response to challenges.

### Reduced Na^+^ influx is responsible for the decreased neuronal excitability in the hippocampus of the neonatally infected group

**Figure 4:**
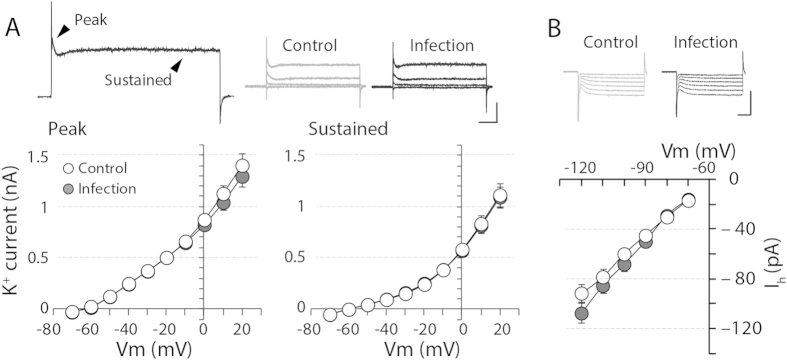
K^+^ channel- and HCN channel- mediated currents are not altered upon neonatal influenza infection. (**A**) A series of voltage steps (500-ms duration, 10-mV increment from −80 to +20 mV) were given in the presence of a mixture of channel inhibitors (CdCl_2_, TTX, PTX, CNQX and AP5). The peak and sustained currents were measured by quantifying the amount of current (shown with arrows). Both the peak and sustained current through the K^+^ channels were comparable in the infected and control groups (n = 28–30, p > 0.4; scale bar: 1 nA and 100 ms). (**B**) A series of hyperpolarizing voltage steps were given to measure I_h_ current through the HCN channel in the presence of the channel inhibitor mixture. There was no significant difference in the amount of I_h_ current between both groups of animals (n = 25–27, p > 0.1; scale bar: 200 pA and 100 ms).

The combinatory action of the voltage-gated channels determines the fine-tuning of the neuronal electrical signals as well as neuronal excitability. K^+^ channels prvide an inhibitory driving force: therefore, the enhanced conductance or number of K^+^ channels would contribute to decreased neuronal excitability. In the hippocampus, more than 35 different primary K^+^ channel subunits are expressed^26^. Therefore, to address any changes in the different types of K^+^ channels, we measured the amplitude of the transient peak current as well as the sustained current upon 500-ms voltage steps (−70 to 20 mV, 10-mV increment) in the presence of 200 μΜ CdCl_2_, 10 μΜ CNQX, 50 μΜ AP5, 100 μΜ PTX and 1 μΜ TTX. The neonatally infected group exhibited comparable amounts of both transient and sustained K^+^ currents to the control group (Fig. 4A, p > 0.4). We then investigated whether another negative regulator channel, a hyperpolarization-activated cyclic nucleotide-gated (HCN) channel, may exhibit functional abnormalities after neonatal infection. The hippocampus expresses a substantial number of HCN channels, which are known to modulate neuronal excitability^27^,^28^,^29^. When we measured the HCN channel-mediated current (I_h_) by giving a series of 500-ms hyperpolarization steps (−70 to −130 mV, 10-mV increment), the amplitude of I_h_ was not significantly enhanced after neonatal influenza infection (Fig. 4B, p > 0.1).

**Figure 5:**
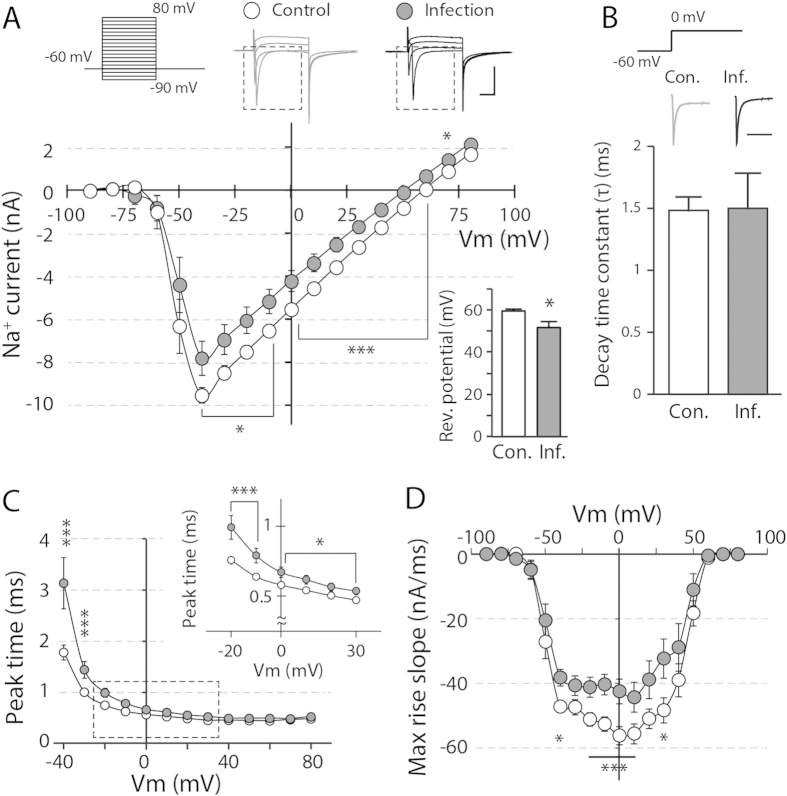
Reduced Na^+^ current upon neonatal influenza infection is responsible for decreased neuronal excitability. (**A**) The current through the voltage-gated Na^+^ channels was measured by giving a series of voltage steps (−90 mV to +80 mV, 10-mV increase each steps) in the presence of CdCl_2_, ZD7288, PTX, CNQX and AP5 in the aCSF, and TEA was added to the internal solution. The Na^+^ channel current was significantly reduced at slightly depolarized potentials (n = 13–18, *p < 0.05, scale bar: 5 nA and 5 ms), leading to a rightward shift in the I-V curve. (Inset) The measured reversal potential of I_Na_ was significantly hyperpolarized in the hippocampal neurons of the infected group (*p < 0.05). (**B**) The inactivation kinetics of the Na^+^ influx remained comparable between the two groups when we compared the decay time constants (n = 13–18, p > 0.9, scale bar: 5 ms). (**C**) The peak time of the Na^+^ channel-mediated currents at each voltage-step was notably delayed in CA1 neurons upon neonatal influenza infection. The inset represents the peak time between −20 and 30 mV (*p < 0.05, ***p < 0.01). (**D**) The max rise slope of I_Na_ was also significantly decreased in the infected group (*p < 0.05, ***p < 0.01).

Neuronal excitability also largely depends on the function of the Na^+^ channels. Thus, we measured the Na^+^ currents (I_Na_) by giving a series of voltage steps (−90 mV to +80 mV, 10-mV increment, 500 ms, activation step protocol) in the presence of 200 μΜ CdCl_2_, 10 μΜ CNQX, 50 μΜ AP5, 100 μΜ PTX and 50 μΜ ZD7288. We found that the Na^+^ channel-mediated current was remarkably reduced in the neonatally infected group in response to larger depolarizations compared with the control group (Fig. 5A, after −40 mV, p < 0.05; after 0 mV, p < 0.01). Tetrodotoxin (TTX) completely abolished these currents when applied at the end of the recordings., The reversal potential for I_Na_ was also significantly hyperpolarized by ~8 mV in hippocampal neurons of the neonatally infected group (Fig. 5A inset, p < 0.05). To further investigate the activation kinetics of TTX-sensitive Na^+^ channels, we analyzed the peak time and maximum rise slope of I_Na_ obtained during the activation step protocol. The peak time of I_Na_ was significantly delayed and the maximum rise slope was smaller in the infected group compared with the control group (Fig. 5C,D, p < 0.05), suggesting that Na^+^ channel opening became notably slower after neonatal infection.

### Steady-state inactivation kinetics of the Na^+^ channel is not different after neonatal influenza infection

**Figure 6:**
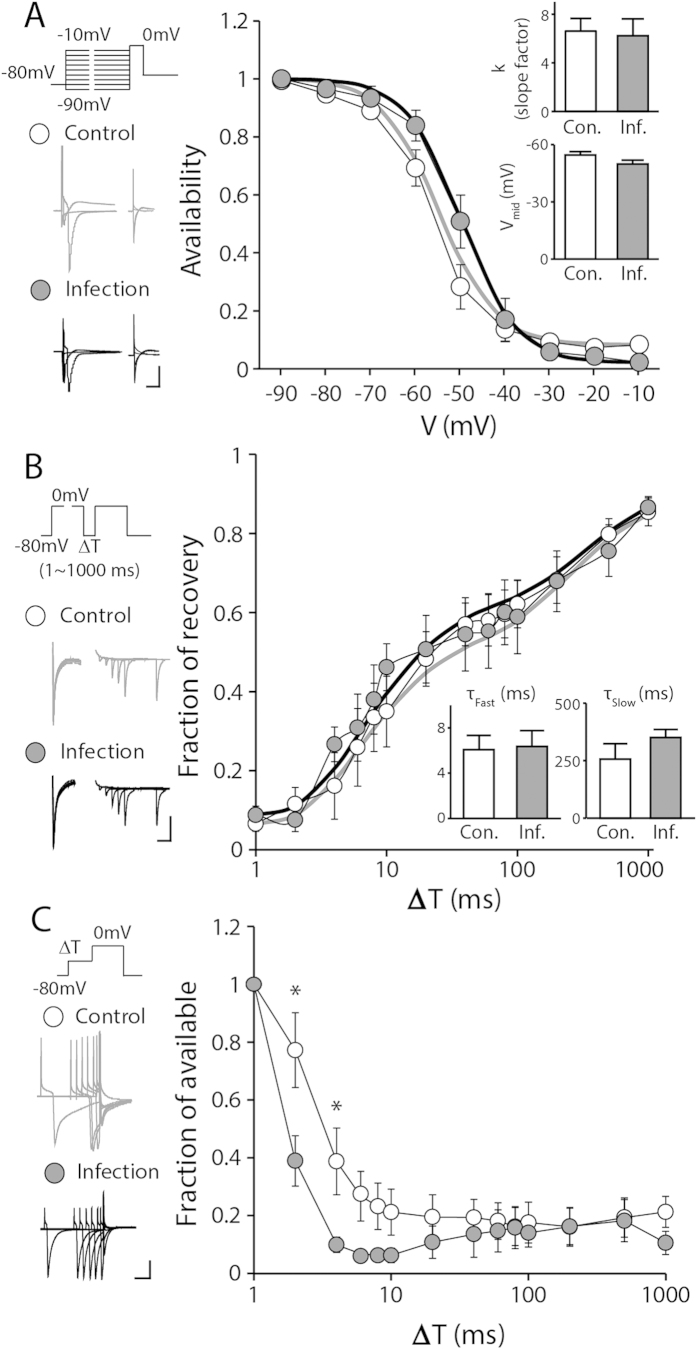
Na^+^ channel inactivation kinetics of the neonatal influenza infected group remains comparable to the uninfected control group. (**A**) The availability was measured by the conductance ratio (G/G_max_) at a test step (0 mV, 30 ms) after pre-inactivation steps (–90 mV to 10 mV, 500 ms, 10-mV increment). The availability of the Na^+^ channels remained comparable in both groups. The median voltage of availability (V_mid_) and the slope factor (*k*) were not altered upon neonatal influenza infection (inset, n = 5–9, p > 0.1, representative traces at –90, –50 and 10 mV step; scale bar: 2 nA and 50 ms). Representative fitting functions are shown in bold lines (*Boltzmann function*, gray: infected control with V_mid_ = –54.86 and *k* = 6.43; black: neonatal infected group with V_mid_ = –49.76 and *k* = 6.24). (**B**) The time course of recovery after inactivation remained intact in the infected group was (ΔT : 1 ms ~ 1000 ms). When the fraction of the recovery plot was fitted with a double-exponential function, both time constants (τ_fast_ and τ_slow_) were not different between two groups (n = 6–7, p > 0.2; scale bar: 250 pA and 2.5 ms). Representative fitting functions are shown in bold lines (*Double-exponential function*; gray: uninfected control with τ_fast_ = 6.08 and τ_slow_ = 256.44; black: neonatal infected group with τ_fast_ = 6.36 and τ_slow_ = 350.58) (**C**) The onset of inactivation was measured with a pre-pulse of increasing duration at −50 mV. The inactivation started significantly earlier at relatively brief pre-pulses in the infected group (ΔT: 2 and 4, *p < 0.05). Beyond 4 ms, the fraction of available Na^+^ channels in the infected group was not different from the control group (n = 6–7, p > 0.06; scale bar: 1 nA and 2.5 ms).

One key feature of Na^+^ channels is their fast inactivation, which largely contributes to the generation of action potentials and neuronal excitability. Slower inactivation of Na^+^ channels would limit the availability of Na^+^ channels in firing, and would thus contribute to less faithful action potential generation in response to a given step-current injection. When we fitted the decay phase of I_Na_ upon the depolarization step from −60 mV to 0 mV shown in Fig. 5A with a single exponential function, there was no significant difference in the decay time constant between the two groups (inactivation time constant τ_ina_ = 1.48 and 1.5, control and neonatally infected group, respectively; Fig. 5B, p > 0.9). Therefore, we aimed to investigate whether neonatal infection would have had an influence on any phases of the inactivation of TTX-sensitive Na^+^ channels with a series of experiments. First, to examine the alterations in voltage-dependent steady-state inactivation, we employed a pre-pulse step protocol that consisted of 500-ms pre-pulses ranging from −90 mV to −10 mV with a10-mV increment followed by a brief depolarizing test pulse to 0 mV for 30 ms (Fig. 6A). The steady-state inactivation of Na^+^ channels determines Na^+^ channel availability at a given potential. Steady-state inactivation was quantified as the ratio of peak I_Na_ at a given pre-pulse potential to the maximum I_Na_, and the ratio was plotted against pre-pulse potentials. The availability plots can be fitted to a single Boltzmann function as described in Materials and Methods. We found no significant difference in either V_mid_ or the slope factor *k* between the two groups, suggesting that there is no major difference in voltage-dependent steady-state inactivation (Fig. 6A, p > 0.9 and p > 0.1 for *k* and V_mid_, respectively).

### Reactivation process of the Na^+^ channel is minimally disturbed after neonatal influenza infection

Na^+^ channels undergo rapid inactivation. Thus, slower recovery from this inactivation may contribute to less faithful action potential firing upon highly challenging stimulations. To examine this possibility, we employed a double-pulse protocol that consisted of a brief 30-ms depolarizing step preceded by a 500-ms depolarizing pre-pulse with variable intervals (Fig. 6B). The peak I_Na_ amplitude evoked by the second depolarization was normalized by the peak amplitude of I_Na_ evoked by the first depolarization. The ratio of the peak I_Na_ amplitudes or the fraction of recovery was plotted against the time interval between the two depolarizing steps (Fig. 6B). When the fraction of recovery plots were fitted to a double-exponential function, we found no significant difference in either the fast or slow time constants between the neonatally infected and control groups (Fig. 6B, p > 0.8 for τ_fast_ and p > 0.2 for τ_slow_), suggesting that the Na^+^ channel reactivation kinetics remain comparable after neonatal influenza infection.

### Inactivation of the Na^+^ channels at sub-threshold voltages are facilitated in the hippocampus of neonatally infected animals

As no major alterations in steady-state inactivation or the recovery from inactivation were observed, we investigated whether influenza infection would have affected Na^+^ channel inactivation at sub-threshold voltages. We measured the onset of inactivation with a pre-pulse protocol that consisted of a pre-pulse with increasing durations at −50 mV followed by a voltage step to 0 mV for 30 ms (Fig. 6C). The peak amplitudes of I_Na_ evoked after a pre-pulse with various durations were normalized to the peak I_Na_ that was obtained without a pre-pulse. There was a significant difference in the inactivation at sub-threshold voltages between the two groups when the interval between pre-pulse and test pulse was 2 or 4 ms (Fig. 6C, p < 0.05). This observation suggests that spontaneous sub-threshold depolarization may limit the availability of Na^+^ channels in the infected group, thereby causing failures in faithful action potential firing.

Finally, we examined whether slight but significant alterations in Na^+^ channel kinetics may have caused changes in a single action potential after neonatal infection. When we compared the physiological properties of an action potential generated upon a brief 2-ms current injection, neonatal infection did not seem to cause any kinetic changes in the action potential such as in the amplitude or half-width (Table 1, p > 0.5). However, when we analyzed the physiological parameters of the first action potentials generated upon a 500-ms long depolarization, we found that neonatal infection indeed increased the half-width and decreased both the rise and decay slopes of the action potential (Table 2, p < 0.05). This observation demonstrates how the altered kinetics of the Na^+^ channels contributes to the kinetic changes in a single action potential in the infected group.

## Discussion

In the current study, we investigated synaptic deficits in an animal model of neonatal influenza A infection. Infection was induced at a time during which synapse formation and myelination occurs^30^. We showed that neonatal influenza infection causes unfaithful action potential firing upon repeated strong depolarizations and slightly decreases spontaneous transmission. In addition, we found that neonatal influenza infection mainly impairs the activation kinetics of TTX-sensitive Na^+^ channels but causes no major alterations in the inactivation kinetics. However neonatal infection may expedite the inactivation of Na^+^ channels at sub-threshold voltages, thereby further contributing to less faithful action potential firing in response to a large depolarization.

We observed that neonatal influenza A virus infection caused mild impairments in key components of the information processing of the hippocampus. First, a slight decrease in the frequency of sEPSCs in the hippocampus was observed after neonatal infection. This observation is consistent with previous studies that have reported a selective decrease in sEPSCs frequency in cultured hippocampal neurons after a brief exposure to influenza ribonucleoprotein (RNP) and NP^19^,^20^. Therefore, influenza virus protein seems to remain in the neonatal hippocampal neurons long after the infection and induces deficits in the basal synaptic transmission. However, this observation could be due to neuronal death, as neonatal infection has been shown to decrease cell viability^25^. Thus, fewer surviving neurons after neonatal infection exhibited lower network activity compared with the control group.

We observed that evoked synaptic transmission was less faithful when challenged with repeated stimulations, whereas we found no major deficits in the pre- or postsynaptic components of the hippocampus in the neonatally infected group. Notably, the ratio of AMPAR- and NMDAR-mediated currents remained unchanged. NP co-localizes with actin filaments, which bind to AMPARs or NMDARs in the dendritic spines^19^,^20^. Moreover, hemagglutinin, which is found on the influenza virus surface, was shown to fuse with the C-terminus of AMPARs^31^. These previous results suggest that the lingering influenza proteins may have an impact on the expression of AMPARs and NMDARs. However, the infection-induced regulations of these glutamate receptors may not be the primary mechanism. In our study, we did not observe any functional changes in these receptor-mediated currents. These observations suggest that although infected hippocampal neurons may have minimal deficits at rest, they may not perform reliable synaptic transmission under highly challenging circumstances, such as successive electrical stimulations. The imperfect synaptic transmission upon repeated challenges after neonatal infection may possibly be due to either a slower recovery from the previous round of neurotransmission or a shortage in synaptic players that have been saved for excessive usage. We further identified that the hippocampal neurons of infected animals also fire less action potentials upon a large depolarization. Taken together, our observations suggest that infected yet surviving mice exhibit mild impairments in mediating basal synaptic transmission. However, the neonatal infection may have impaired the synaptic efficacy required to accommodate highly challenging conditions such as successive stimulations at high frequency or large depolarization.

What is the underlying mechanism responsible for the deficits in neurotransmission in the infected yet surviving animals? We found that their threshold to initiate action potentials or resting membrane potential remained comparable to the control group, suggesting minimal disturbances in the function of K^+^ channels. Indeed, we observed no significant difference in K^+^ channel-mediated currents between the two groups of animals. However, the Na^+^ influx in the hippocampus of the infected yet surviving animals was remarkably decreased compared with controls. The decrease in transient I_Na_ that we observed (~20%) is comparable to the amount of Na^+^ current reduction that occurs in the aged hippocampus^32^. We showed that the reversal potential of the Na^+^ channel was far more hyperpolarized in the neonatally infected group, thereby decreasing the driving force for I_Na_. It is possible that mechanical defects in the opening and/or closing of the Na^+^ channels may have caused less firing upon depolarization as well as set a hyperpolarized reversal potential. Our thorough examination of both the activation and inactivation kinetics of the Na^+^ channels with various step protocols revealed that neonatal infection caused slower activation of the Na^+^ channels, which lead to decreased Na^+^ influx upon a large depolarization with no major defects in the inactivation phases. Although the inactivation phase of the Na^+^ channels after neonatal infection remained largely comparable to the control group, the sub-threshold inactivation have been facilitated after neonatal infection. The facilitated sub-threshold inactivation may contribute to limit the availability of the Na^+^ channels, leading to reduced Na^+^ influx in the neonatally infected group. Taken together, our observations suggest that the driving force of I_Na_ would have decreased possibly due to the changes in Na^+^ concentrations across the membrane. In addition, I_Na_ *per se* would have been reduced primarily due to the impaired activation of Na^+^ channels, thereby causing failures in action potential firing upon a large depolarization. A few previous studies have shown that the influenza virus inhibited the function and decreased the expression level of Na^+^ channels during the replication period^33^,^34^. In addition, M2 influenza virus protein has been shown to interact with the Na^+^ channel and activate protein kinase C (PKC) -dependent signaling pathways in epithelial cells^35^.

Our study is the first to demonstrate alterations in Na^+^ influx after neonatal influenza infection in neuronal preparations. Other regulators of neuronal excitability such as K^+^ channels and HCN channels seem to function within normal ranges. These mice were neonatally infected yet they survived. A K^+^ channel malfunction, for example, may have caused more severe deficits during early development, thereby leading to a possibly lethal condition. The mechanisms underlying the alterations in the voltage-dependence of Na^+^ conductance after neonatal infection remain to be investigated. Further studies are also required to elucidate how decreased neuronal excitability may lead to mental disorders such as autism and schizophrenia^15^. Our current study provides physiological insights into understanding influenza infection-associated functional changes in synapses and perhaps the predisposition to psychiatric and mental disorders upon neonatal infection.

## Materials and Methods

### Animals and virus infection

Five-day-old Balb/c mice with an average body weight of 2.8 g were infected intraperitoneally with mouse-adapted neurotropic influenza A virus (H1N1, A/NWS/33, ATCC VR-219) at a dose of LD_50_ (10^3.5^ TCID_50_/mL) as described previously^25^. Animals were monitored daily after influenza infection until their use in the study. Mice that survived until 21 days post-infection (dpi) and age-matched control mice were used in this study. All mice were maintained under a 12:12 h light:dark cycle. Food and water were provided ad libitum until the time of the experiments. Throughout the protocols, all efforts were made to minimize the number of animals used and their suffering. During this study, humane endpoints were applied in accordance with Konkuk University’s animal experiment protocols. The Institutional Animal Care and Use Committee at Konkuk University approved the experimental protocols for the care and use of laboratory animals (KU10051). The animal experimental methods were carried out in accordance with the approved protocol.

### Slice preparation

Male mice (24–30 days old) were used for all the electrophysiology experiments. Animals were anaesthetized briefly with isoflorane, and after immediate decapitation their brains were stored and dissected in ice-cold dissection buffer (in mM, 212 sucrose, 3 KCl, 26 NaHCO_3_, 1.25 NaH_2_PO_4_, 7 MgCl_2_, and 10 glucose, gassed with 95% O_2_ and 5% CO_2_). Coronal slices (350-μm thick) containing the hippocampus were prepared using a VT 1000S vibratome (Leica, Germany). Brain slices were then transferred to a recovery chamber containing artificial cerebrospinal fluid ([aCSF], in mM, 118 NaCl, 2.5 KCl, 1 NaH_2_PO_4_, 26.2 NaHCO_3_, 1 MgCl_2_, and 2 CaCl_2_, gassed with 95% O_2_ and 5% CO_2_) at 35 °C for an hour before recording.

### Electrophysiology

Experiments were performed on the interleaved wild-type control and infected animals. Experimenters remained blinded to the experimental group. CA1 pyramidal neurons were voltage clamped at −60 mV using either Axopatch 200B and Clampex 10.3 (Molecular Devices, USA), or HEKA EPC8 and pulse v8.8 (HEKA electronic, Germany), filtered at 5 kHz, and sampled at 10 kHz at room temperature. Glass pipettes with a resistance of 2–6 MΩ were used. The internal solution for the voltage clamp recording contained the following (in mM): 115 Cs methanesulphonate, 20 CsCl, 10 HEPES, 2.5 MgCl_2_, 0.6 EGTA, 5 lidocaine N-ethyl bromide, 4 Na_2_-ATP, 0.4 Na_2_-GTP, and 10 Na-phosphocreatine. The internal solution for the current clamp recording contained the following (in mM): 130 K-gluconate, 10 HEPES, 0.6 EGTA, 5 KCl, and 2.5 Mg-ATP.

To record EPSCs (excitatory postsynaptic currents), picrotoxin (PTX, 50 μM in DMSO) was added to the aCSF to exclude GABAR-mediated synaptic transmission. Evoked EPSCs (eEPSCs) were measured by a stimulator, placed on the Schaffer collateral pathway. Responses were recorded at holding potentials of −60 mV (for AMPAR-mediated responses) and +40 mV (for NMDAR-mediated responses). NMDAR-mediated responses were quantified as the amplitude at 75 ms after stimulation to exclude a possible AMPAR-mediated eEPSC contribution. Spontaneous EPSCs (sEPSCs) were recorded in the same condition without stimulation. sEPSC events were analyzed manually to avoid false-positive and false-negative events using Mini Analysis software (Synaptosoft, USA).

The internal solution for measuring K^+^ currents and I_h_ contained the following (in mM): 130 K-gluconate, 10 HEPES, 0.6 EGTA, 5 KCl, and 2.5 Mg-ATP. The internal solution for measuring Na^+^ currents contained the following (in mM): 20 TEA, 145 CsCl, 10 HEPES, 10 EGTA, 2 NaCl, and 2 Mg-ATP. For most Na^+^ current recordings, the built-in circuit of the Axopatch 200B amplifier (up to 80% prediction and 70% correction) compensated the series resistance. The liquid junction potential was measured to +2.4 mV and was not corrected during data acquisition.

### Data analysis

The decay time constant of Na^+^ influx was calculated by fitting the I_Na_ with a single exponential function starting at the peak of the amplitude. The availability of the Na^+^ channels after the pre-voltage steps was calculated as a conductance ratio (G/G_max_) which was calculated by Ohm’s law:


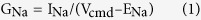


where V_cmd_ is the command voltage and E_Na_ is the estimated reversal potential of Na^+^ channels in mV. The pre-voltage steps were 500 ms long and varied from −90 mV to −10 mV with a 10-mV increment. The availability plot was fitted to a single Boltzmann function:


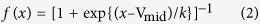


where V_mid_ is the half-inactivation potential and *k* is the slope factor. The fraction of recovery was calculated by I_Δt_/I_max_ (I_Δt_: the second peak amplitude after Δt; I_max_: the first peak amplitude during 500-ms pre-pulse at 0 mV). The fraction of recovery plot was fitted to a double-exponential function:


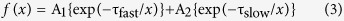


The fraction of availability was measured by I_Δt_/I_max_ (I_max_: the largest amplitude during a test pulse) and plotted against Δt an the x-axis. The values are given as the mean ± SEM and two-tailed unpaired t-tests were used for statistical comparisons unless stated otherwise.

### Protein extraction and western blot analysis

Four mice per group were anesthetized with intraperitoneal injections of zoletil (90 mg/kg) and xylazine (10 mg/kg), and their hippocampi were collected. The extracted proteins were quantified by the BCA Protein Assay System (Pierce, USA). The proteins were subjected to SDS-PAGE, followed by electro-transfer to PVDF membranes (Millipore, USA). After blocking with 5% skim milk in 0.1% Tris-buffered saline (TBS)/Tween-20, the blots were probed with primary antibodies against GluA1 (1:1000, Abcam), GluN1 (1:1000, Sigma-Aldrich), GluN2A (1:2000, Millipore), NR2B (1:500, BD Transduction Laboratories), and synaptophysin (1:4000, Abcam) overnight at 4 °C. After incubation, the membranes were subsequently incubated for 2 h at room temperature with the appropriate secondary antibodies conjugated with horseradish peroxidase (Vector, USA). The proteins were visualized with an enhanced chemiluminescence kit (Pierce, USA) and band intensities were quantified using the Kodak Gel Logic 2200 imaging system with Molecular Image analysis software (Kodak, USA). β-Actin was used as a loading control (1:10000, Sigma-Aldrich). Data are presented as an average of at least three experiments and analyzed for statistical significance by using Mann-Whitney tests (Prism; Graph Pad, USA).

## Additional Information

**How to cite this article**: Park, H. *et al*. Decreased Na^+^ influx lowers hippocampal neuronal excitability in a mouse model of neonatal influenza infection. *Sci. Rep*. **5**, 13440; doi: 10.1038/srep13440 (2015).

## Figures and Tables

**Table 1:** Comparison of the physiological parameters of an action potential during brief depolarization between uninfected and neonatally-infected hippocampal neurons.

	Uninfected control	Neonatal infection	*p* value
Max rise slope (dV/dt)	160.87 ± 21.35	173.7 ± 18.13	0.662
Max decay slope (dV/dt)	−107.25 ± 9.46	−100.71 ± 3.78	0.559
AP half-width (ms)	1.6 ± 0.1	1.64 ± 0.08	0.781
AP amplitude (mV)	120.27 ± 4.86	120.07 ± 2.8	0.531

The values represents the mean ± SEM (n = 6–7). Student’s two tailed t-test: *p < 0.05, ***p < 0.01.

**Table 2:** Comparison of physiological parameters of an action potential during a 500-ms depolarization between uninfected and neonatally-infected hippocampal neurons.

	Uninfected control	Neonatal infection	*p* value
Max rise slope (dV/dt)	219.89 ± 34.26	124.23 ± 20.32	0.024*
Max decay slope (dV/dt)	−102.22 ± 12.51	−57 ± 9.84	0.011*
AP half-width (ms)	1.00 ± 0.06	1.81 ± 0.23	0.008***
AP amplitude (mV)	80.28 ± 5.36	76.09 ± 6.11	0.626
Number of spikes induced by a 500-ms step current injection of 300 pA	13.24 ± 1.54	9.44 ± 1	0.03*

The values represent the mean ± SEM (n = 7–9). Student’s two tailed t-test: *p < 0.05, ***p < 0.01.
